# Chinese herbal medicine combined with entecavir to reduce the off-therapy recurrence risk in HBeAg-positive chronic hepatitis B patients: a multicener, double-blind, randomized controlled trial in China

**DOI:** 10.1186/s13063-020-04417-9

**Published:** 2020-08-12

**Authors:** Xiaoke Li, Ludan Zhang, Mei Qiu, Yi Huang, Huanming Xiao, Bingjiu Lu, Yuyong Jiang, Fuli Long, Hui Lin, Jinyu He, Qikai Wu, Mingxiang Zhang, Li Wang, Xiaoning Zhu, Man Gong, Xuehua Sun, Jianguang Sun, Fengxia Sun, Wei Lu, Weihua Xu, Guang Chen, Zhiguo Li, Danan Gan, Xianzhao Yang, Hongbo Du, Yong’an Ye

**Affiliations:** 1 Institute of Liver Diseases, Beijing University of Chinese Medicine, Dongzhimen Hospital affiliated to Beijing University of Chinese Medicine, Beijing, 100700 China; 2 Department of Hepatology, Shenzhen Traditional Chinese Medicine Hospital, Shenzhen, 518033 Guangdong Province China; 3 Department of Hepatology, Chongqing Traditional Chinese Medicine Hospital, Chongqing, 400021 China; 4 Department of Hepatology, Guangdong Hospital of Traditional Chinese Medicine, Guangzhou, 510006 China; 5 Department of Hepatology, Liaoning Hospital of Traditional Chinese Medicine, Shenyang, 110032 China; 6 Department of Hepatology, Beijing Ditan Hospital, Beijing, 100015 China; 7 Department of Hepatology, The First Affiliated Hospital of Guangxi University of Chinese Medicine, Nanning, 530023 China; 8 Department of Hepatology, Mengchao Hepatobiliary Hospital of Fujian Medical University, Fuzhou, 350025 China; 9 Department of Hepatology, Shaanxi Hospital of Traditional Chinese Medicine, Xi’an, 710003 China; 10 Department of Hepatology, The Third People’s Hospital of Shenzhen, Shenzhen, 518112 Guangdong Province China; 11 Department of Hepatology, The Sixth People’s Hospital of Shenyang, Shenyang, 110006 China; 12 Department of Hepatology, Public Health Clinical Center of Chengdu, Chengdu, 610066 China; 13 Department of Hepatology, Affiliated traditional Chinese Medicine Hospital of Southwest Medical University, Luzhou, 646699 China; 14 Department of Hepatology, 302 Military Hospital of China, Beijing, 100039 China; 15 Department of Hepatology, Shanghai Shuguang Hospital, Shanghai, 200021 China; 16 Department of Hepatology, Shandong Hospital of Traditional Chinese Medicine, Jinan, 250011 China; 17 Department of Hepatology, Beijing Chinese Medicine Hospital, Beijing, 100010 China; 18 Department of Hepatology, The Second People’s Hospital of Tianjin, Tianjin, 300000 China; 19 Department of Gastroenterology, The Second Hospital of Shandong University, Jinan, 250100 China

**Keywords:** Chronic hepatitis B, Chinese herbal medicine formula, Protocol, Randomized-controlled trial

## Abstract

**Background:**

Nucleos(t)ide analogues (NAs) are the first-line option against chronic hepatitis B (CHB). NAs produce potent suppression of viral replication with a small chance of HBsAg seroclearance and a high risk of virological relapse after discontinuation. The combined therapy of NAs plus traditional Chinese medicine (TCM) is widely accepted and has been recognized as a prospective alternative approach in China. Based on preliminary works, this study was designed to observe the therapeutic effect of TCM plus entecavir (ETV) against HBeAg-positive chronic hepatitis B with respect to reducing the recurrence risk after NA withdrawal.

**Methods/design:**

The study is a nationwide, multicenter, double-blind, randomized, placebo-controlled trial with a duration of 120 weeks. A total of 18 hospitals and 490 eligible Chinese HBeAg-positive CHB patients will be enrolled and randomly allocated into the experimental group and control group in a 1:1 ratio. Patients in the experimental group will be prescribed TCM formulae (Tiaogan-BuXu-Jiedu granules) plus ETV 0.5 mg per day for consolidation therapy for 96 weeks. Patients in the control group will be prescribed TCM granule placebo plus ETV 0.5 mg per day for the same course. After consolidation therapy, all patients will discontinue their trial drugs and be closely monitored over the next 24 weeks. Once clinical recurrence (CR) occurs, ETV treatment will be restarted. The primary outcome is the cumulative rate of CR at the end of this trial.

**Conclusion:**

This study is the first of its kind to observe therapeutic effects with respect to reducing recurrence after NA withdrawals after unified integrative consolidation therapy in the CHB population.

**Trial registration:**

Chinese Clinical Trial Registry No. ChiCTR1900021232. Registered on February 2, 2019

Chronic hepatitis B (CHB) has been one of the most concerning diseases worldwide. Every year, approximately 1 million people die of CHB-related cirrhosis and hepatocellular carcinoma [1]. Nucleos(t)ide analogues (NAs) therapy is widely applied in patients with chronic hepatitis B virus (HBV) infection. Currently, six NAs are available against CHB: lamivudine, telbivudine, entecavir (ETV), adefovir, tenofovir disoproxil fumarate (TDF), and tenofovir alafenamide (TAF), of which ETV and TDF are recommended as first-line therapies [2–4]. NAs inhibit the reverse transcriptase activity of the HBV polymerase and thus suppress viral replication. However, NAs exert little effect on viral covalently closed circular DNA (cccDNA) in the nucleus [5]. Hence, they cannot permanently eradicate the virus. Currently, hepatitis B surface antigen (HBsAg) loss is the most widely accepted endpoint to guide cessation of NA treatment. However, as HBsAg loss is an infrequent event, this strategy entails an indefinite therapeutic duration that could be lifelong for the vast majority of NA-treated patients [6]. Therefore, the benefit of long-term treatment should be weighed against the burden of life-long medication, monitoring, and adherence. In addition, the long-term risk for the development of resistance and adverse events (AEs) remains unclear.

In recent years, some scholars have attempted to find approaches for preventing relapses after NAs withdrawal [7–9]. A previous study observed the outcomes after the cessation of ETV therapy among patients who fulfilled the stopping rules of the Asia-Pacific Association for the Study of the Liver (APASL) [10]. The 2-year cumulative rates of virological and clinical relapse were 41.3% and 33% in hepatitis B e antigen (HBeAg)-positive patients, and the 3-year cumulative rates of virological and clinical relapse were 62.7% and 48.3% in HBeAg-negative patients, respectively. The risk after discontinuation of the therapy is concerning.

Traditional Chinese medicine (TCM) therapy has a long history and definite curative effect for treating various chronic liver diseases including chronic hepatitis and fibrosis/cirrhosis [11–13], and abundant data and experience have accumulated in long-term clinical practice and scientific research. Although TCM therapy alone has no explicit antiviral effect, it has some advantages in improving clinical symptoms [14], alleviating liver inflammation, anti-fibrosis [15], and regulating immune function [16]. In China, supported by major national special research projects funded by the Ministry of Health, some research results have been obtained regarding the treatment of chronic hepatitis B by the combination of TCM and NAs, the advantages of TCM therapy in the treatment of chronic hepatitis B have been preliminarily clarified [17], an integrated TCM and NAs therapeutic schedule has been developed [18], and it has been proven that the combination of TCM and NAs can significantly enhance the negative conversion ratio of HBeAg, including that seen in refractory diseases [19, 20]. Based on the preliminary work, this study carries out research on combination TCM and NAs strategies for HBeAg-positive chronic hepatitis B. Through a national multicenter double-blind, randomized controlled study, we will verify the effect of combination TCM and NA therapy in reducing the recurrence rate after drug withdrawal.

## Registration

We had registered this trial before recruitment on the Chinese Clinical Trial Registry (No. ChiCTR1900021232). This trial will be conducted following the principles of the Declaration of Helsinki (2004 version). The study protocol has been approved by the Ethics Committee of Dongzhimen Hospital affiliated to Beijing University of Chinese Medicine before recruitment (Approval number: DZMEC-KY-2018-61).

## Recruitment

A total of 490 patients will be recruited by nineteen clinical centers in China nationwide listed as follows: Dongzhimen Hospital affiliated to Beijing University of Chinese Medicine, Shenzhen Traditional Chinese Medicine Hospital, Guangdong Hospital of Traditional Chinese Medicine, Liaoning Hospital of Traditional Chinese Medicine, the First Affiliated Hospital of Guangxi University of Chinese Medicine, Shaanxi Hospital of Traditional Chinese Medicine, Mengchao Hepatobiliary Hospital of Fujian Medical University, Beijing Chinese Medicine Hospital, Beijing Ditan Hospital, Chongqing Traditional Chinese Medicine Hospital, Affiliated traditional Chinese medicine hospital of Southwest Medical University, Shanghai Shuguang Hospital, the Sixth People’s Hospital of Shenyang, 302 Military Hospital of China (the Fifth Medical Center of People’s Liberation Army of China), Shandong Hospital of Traditional Chinese Medicine, the Second People’s Hospital of Tianjin, Public Health Clinical Center of Chengdu, the Third People’s Hospital of Shenzhen, and the Second Hospital of Shandong University. We will recruit volunteers from the outpatient department of the hospitals.

The principal investigators (PIs) of subcenters will introduce the protocol as well as the benefits and risks of the study to the participants before enrollment. In the informed consent form, participants will be asked if they agree to allow the use of their data should they choose to withdraw from the trial. Participants will also be asked for permission for the research team to share relevant data with people from the universities taking part in the research or from regulatory authorities, where relevant. This trial does involve collecting biological specimens for storage. There is no anticipated harm from or compensation for trial participation.

The inclusion and exclusion criteria are listed below. The criteria for premature withdrawal from the study include protocol deviation, pregnancy, or investigator discretion.

## Inclusion criteria

The inclusion criteria are as follows: (1) diagnosed CHB with positive HBeAg, who has experienced HBeAg clearance and/or seroconversion; (2) aged between 18 and 65; (3) TBIL < 3 × ULN; (4) HBsAg < 5000 IU/ml; (5) TCM symptoms are classified as stagnation of the liver and deficiency of Spleen Qi and Damp-Heat in the liver and gallbladder; and (6) voluntarily sign informed consent.

In the informed consent form, we explain the protocol and clearly inform the participants that after 96 weeks of consolidation therapy, they will stop NA treatment and be closely monitored for the next 24 weeks. Although the procedure complies with the AASLD [21], APASL [22], and EASL [23] guidelines for CHB treatment, there could be potential risks (including virological or clinical relapse) during the off-therapy period, and patients may be required to restart antiviral therapy. Patients are free to quit the trial any time before the off-therapy period.

## Exclusion criteria

Any of the following cases will be excluded: (1) diagnosed with chronic hepatitis caused by non-viral causes, overlapped virus infections, or cryptogenic hepatitis; (2) accompanied by liver failure, hepatocirrhosis (including stage 4 fibrosis), hepatic encephalopathy, electrolyte disorders, gastrointestinal bleeding, fatal infections, or any other severe complications; (3) diagnosed with malignant tumors or with a progressive elevation of serum tumor markers; (4) diagnosed with primary or secondary cardiovascular, cerebrovascular, pulmonary, renal, endocrine, nerve, and hematology diseases; (5) participating or participated in other clinical trials within 1 month; (6) diagnosed mental disorders; (7) confirmed hepatitis B virus pre-C/C or P gene variants; (8) pregnant or lactating women, individuals that are scheduled to conceive or fertilize; and (9) with other unfavorable conditions.

## Sample size estimation

We calculated the sample size with PASS software (NCSS, LLC. Utah, USA. Version 15.0.5). Referencing a study published in 2016, in which the recurrence rate was 32% in HBeAg positive CHB patients after NA withdrawal within 24 weeks [24], we determined that we would need to achieve 80% power to detect a difference between the group proportions of 12%. The required significance level was set to 0.05. We set the sample size to 245 for each group, 490 in total, for which a 20% loss of participants was considered.

## Study drugs

The experimental drug (TCM granules) is Tiaogan-Buxu-Jiedu (TGBXJD) granules. All patients will be randomized to treatment with TGBXJD granules (orally, one dose per day) plus entecavir (ETV 0.5 mg per day) or TGBXJD granule placebo plus ETV. TGBXJD granules (including placebo formulations, 30 g per dose) will be provided by PuraPharm Co. Ltd., China (lot No. A190069710). The manufacturing procedures are as follows: every herb (*Bupleurum*, *Atractylodes* rhizomes, and *Scutellaria* root) will be extracted with 97 ± 2 °C water. The extract will then be filtered through 200 μm mesh filter cloth, and the filtrate will be concentrated under a vacuum to a relative density of 1.10~1.20 (temperature 60 ± 5 °C). The concentrate will be mixed after stirring for 30 min. The herbal granules will be collected with the dry granulation method. ETV tablet (0.5 mg per tablet) will be provided by CHIATAI TIANQING company, China (lotNo. 181214101, 181203201, 181024101). The major ingredients of TGBXJD are *Scutellaria*, *Bupleurum*, and *Atractylodes*.

## Therapeutic regimen

**Fig. 1:**
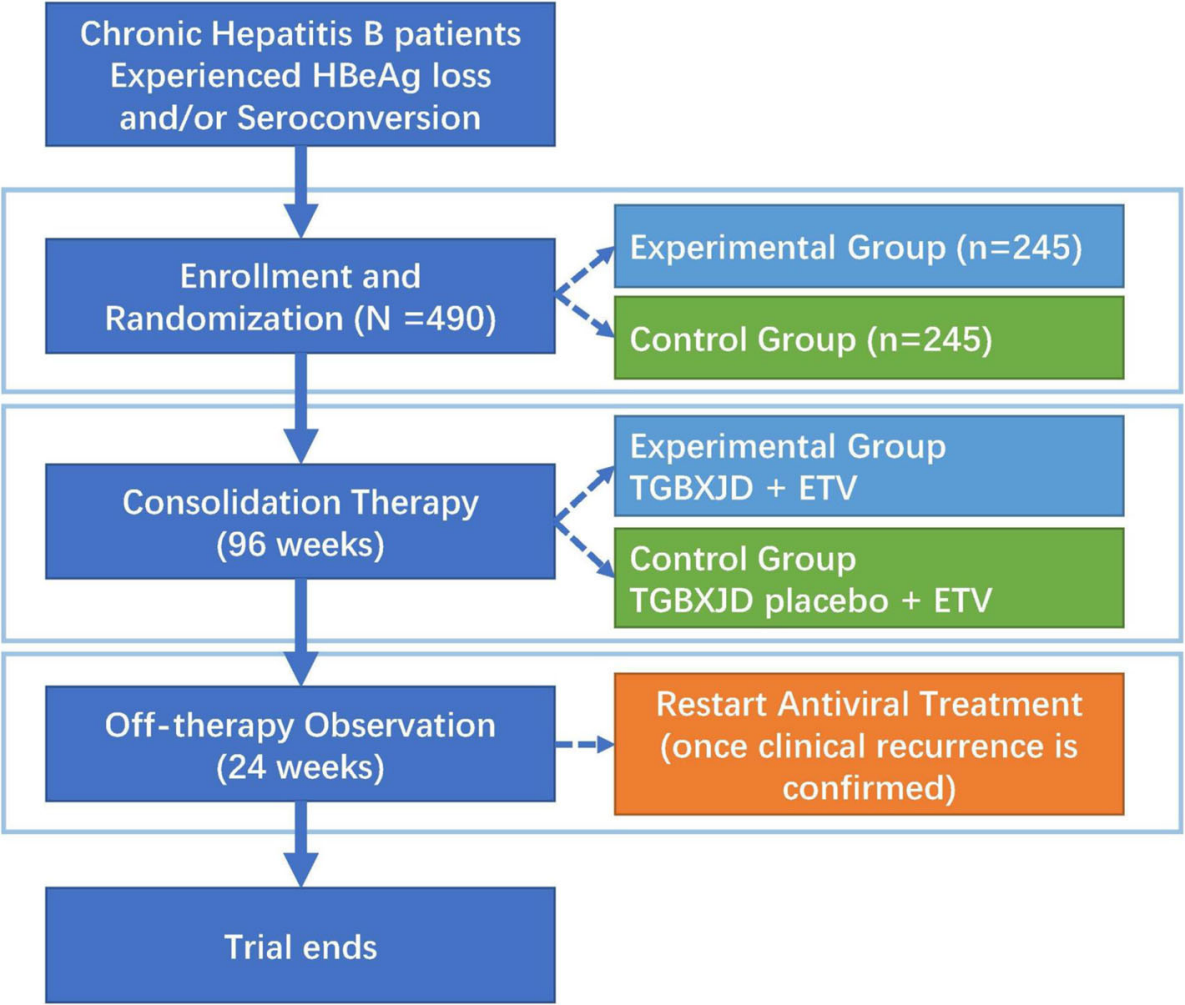
Flowchart of the study design

Patients who meet the inclusion criteria will be allocated in a 1:1 ratio to either the experimental group (EG) or the control group (CG). Patients in the EG will receive TGBXJD granules along with ETV. Patients in the CG will receive TCM placebo along with ETV. The treatment duration is 96 weeks. Those who develop clinical relapse during consolidation therapy (in the first 96 weeks) will be switched to TDF (300 mg per day) according to the guidelines [25] and be removed from the study. All patients who meet the following NA withdrawal criteria in the first 96 weeks will stop their ETV treatment and will be closely monitored in the following 24 weeks: (1) serum ALT persistently within the normal range (under 40 IU/L) and (2) serum HBV DNA persistently undetectable (under 30 IU/mL). Once clinical recurrence is confirmed during the off-therapy period, antiviral treatment (entecavir, 0.5 mg per day) will be restarted immediately (Fig. 1).

## Randomization and blinding

Stratified block randomization will be conducted by the Chinese Academy of Chinese Medicine Sciences (CACMS) with their online central randomization system. Clinical physicians in subcenters will oversee the recruitment process. Each patient will be given a unique ID through a web interface provided by CACMS, where they will be randomly allocated to each group at a ratio of 1:1 via the central randomization system. Each trial drug will be labeled with a unique number and dispatched with the online system at each visit. All experimental drugs and corresponding placebo treatments are consistent in appearance and taste. The grouping information of each participant will be concealed to all research personnel and data analyzers until the trial ends. Unblinding will be permitted in cases of serious AEs.

## Interviews

From the first day to the observation point of week 96, we will follow up patients every 24 weeks (± 3 days) and dispatch trial drugs on each visit. In addition, we will retrieve the empty boxes and the remaining doses of each patient for verification and recording. At the end of week 96, patients will be informed again about the potential risks of stopping NA therapy, and additional consent to participate in off-therapy observation will be required. From week 96 to week 120, we will set the intervals between interviews to 4 weeks (± 3 days).

Specimens collected during the trial will be stored in the Biobank of Dongzhimen Hospital. The specimens will be used to reconfirm the readings of existing assessments if necessary, as well as for genetic or molecular analysis in current trials and for future use in ancillary studies.

## Assessments

To ensure safety and reveal the curative effects of trial drugs, patients will be monitored regularly with assessments throughout the trial (Table 1).

**Table 1:** Scheduled assessments

Assessment	Consolidation therapy									Off-therapy observation						Treatment after recurrence
	Day 1	12 w	24 w	36 w	48 w	60 w	72 w	84 w	96 w	4 w	8 w	12 w	16 w	20 w	24 w	Recurrence
**Blood, urinary, and stool routine test**	**√**		**√**		**√**		**√**		**√**						**√**	
**Liver functions**	**√**		**√**		**√**		**√**		**√**	**√**	**√**	**√**	**√**	**√**	**√**	**√**
**Renal function**	**√**		**√**		**√**		**√**		**√**						**√**	
**HBV-serum marker**	**√**		**√**		**√**		**√**		**√**			**√**			**√**	**√**
**HBV DNA**	**√**		**√**		**√**		**√**		**√**	**√**	**√**	**√**	**√**	**√**	**√**	**√**
**Liver biopsy**	**√**								**√**							
**ccc DNA**	**√**								**√**							
**HBcrAg**	**√**		**√**		**√**		**√**		**√**			**√**			**√**	
**HBV pregenomic RNA**	**√**		**√**		**√**		**√**		**√**			**√**			**√**	
**LHBs**	**√**		**√**		**√**		**√**		**√**			**√**			**√**	
**AFP**	**√**		**√**		**√**		**√**		**√**						**√**	
**PT**										**√**	**√**	**√**	**√**	**√**	**√**	
**ECG**	**√**		**√**		**√**		**√**		**√**						**√**	
**Abdominal ultrasound scan**	**√**		**√**		**√**		**√**		**√**						**√**	
**Antibodies of hepatitis A, C, D, E**	**√**															
**CLDQ**	**√**	**√**	**√**	**√**	**√**	**√**	**√**	**√**	**√**			**√**			**√**	
**SF-36**	**√**	**√**	**√**	**√**	**√**	**√**	**√**	**√**	**√**			**√**			**√**	
**The symptom score of TCM**	**√**	**√**	**√**	**√**	**√**	**√**	**√**	**√**	**√**			**√**			**√**	

Patients will be followed up every 12 weeks during the consolidation therapy and every 4 weeks in the off-therapy observation (“w” is short for “weeks”)

*Abbreviations*: *HBV* hepatitis B virus, *ccc DNA* covalently closed circular DNA, *HBcrAg* hepatitis B core-related antigen, *LHBs* HBV large surface proteins, *AFP* alpha fetoprotein, *PT* prothrombin time, *ECG* electrocardiogram, *SF-36* the Short Form (36-item) Health Survey, *CLDQ* the Chronic Liver Disease Questionnaire

## Primary outcomes

The primary outcome will be the incidence of recurrence after ETV withdrawal. Clinical recurrence is defined as a serum aminotransferase (ALT) over twofold of the upper limit of normal (ULN) while the HBV DNA > 2000 IU/mL.

## Secondary outcomes

Secondary outcome measurements include virus serological indicators, HBV DNA, liver function, liver biopsy results, cccDNA, hepatitis B core-related antigen (HBcrAg), HBV pregenome RNA (pgRNA), HBV large surface proteins (LHBs), the symptom score of TCM, the Short Form (36-item) Health Survey (SF-36), and the Chronic Liver Disease Questionnaire (CLDQ).

## Safety monitoring

Primary vital signs and laboratory parameters will be assessed in patients regularly. Primary vital signs include body temperature, blood pressure, heart rate, and respiratory rate. Laboratory tests include routine blood, urine, and stool tests, along with fecal occult blood tests, ECG, and abdominal B ultrasound scan. In particular, relapse after drug discontinuation is defined as an HBV DNA level > 2000 IU/mL with a serum ALT > 2 × ULN.

## Adverse events

We will document and report any AEs that occur during the trial and analyze any causal relationships between such AEs and the trial drug (TGBXJD granules). If any AE occurs, the PIs will ensure that the participant receives the corresponding treatment. If a relationship between the AEs and the trial drug cannot be ruled out, the event will be reported to the ethical committee and the project supervisor to determine whether or not the treatment should be suspended for the individual.

## Statistics analysis

An independent statistical committee will be in charge of the analysis. The statistical analysis team will conduct the intention-to-treat (ITT) analysis for the primary outcome, where all randomized populations will be included in the full analysis set (FAS). Subjects who violated the protocol will be inspected individually. The last observation carried forward method will be used to impute missing data.

### Missing data

Missing data will be inspected manually; if critical assessments for judging clinical relapse are incomplete (including ALT and HBV DNA) during the off-therapy period, the case will be excluded from the primary outcome analysis. If the incomplete data only affect the secondary or safety analysis, we will impute them with the last observation carried forward method.

### Data description

Normally distributed continuous variables will be expressed as the means ± standard error of the mean (SEM), abnormally distributed continuous variables will be expressed as medians and interquartile ranges, and categorical variables will be expressed as numbers and percentages.

### Outcome analysis

For the primary outcome, the proportions of patients who developed off-therapy relapse in each group will be compared with the Pearson chi-square method. Time to event analysis will be performed with the Kaplan-Meier method. For the secondary outcomes, the data are continuous variables, for which we will use repeated-measures mixed effects models to test differences between the groups. The total effect of the scale scores from questionnaires will be tested with the Wilcoxon rank sum test. A paired *t* test will be used for comparisons of data before and after treatment within groups. A two-sided *p* value < 0.05 will be considered statistically significant; confidence intervals (CIs) are set at 95%.

### Safety analysis

Safety analysis will be performed based on the subjects who actually received treatment. We will compare the data with Student’s *t* test or the Mann–Whitney *U* test. In addition, abnormal results from safety assessments will be further classified as “abnormal (no clinical significance)” and “clinically abnormal.” The latter will be inspected to determine they were caused by the trial drugs. The incidence of AEs will be described, and the specific grades of all the AEs in each group and their relationships with drugs will be described in detail. The proportions of patients who developed AEs between groups will be compared with Pearson’s chi-square test.

## Data management and quality control

Clinical physicians will collect data by means of a standardized paper case report form (CRF). Investigators will record any deviations from the protocol on the breach report form, which is included in the CRF. Paper CRFs will be stored in accordance with national regulations. The electronic MedInform system will provide an online platform for collecting data produced by the trial. The data will be stored in the system’s cloud drives. The website for accessing the system is http://linkermed.com/edc/a. We will employ the Contract Research Organization to acquire, enter, and check results translated from local laboratories to the platform.

During the study, an independent data monitoring committee (DMC) will be set up to carry out periodic interim evaluation and optimize the study when appropriate based on the results of the interim evaluation. The DMC is authorized to discontinue the clinical study in the case of unexpected adverse reactions. During implementation of the project, the original trial data will be subject to audit and random inspection periodically or irregularly. In addition, study compliance will be checked so that data integrity and accuracy will be fully guaranteed and the authenticity and reliability of the study results will be ensured.

A clinical monitor will visit the study sites every 3 months to check the progress of the study. Important points to be checked include whether the investigator has conducted the study as per protocol, how many participants have been screened and enrolled, and if all eligible participants signed the informed consent form. Completeness of the case report form and other essential documents, as well as records of any drop-outs or AEs, will be checked for correctness and consistency with the source documents in a timely manner.

## Protocol amendments

If there are amendments to the protocol, we will deliver a formal memorandum with a revised protocol to the funder and ethical committee. PI will notify the investigators of all subcenters and send a copy of the revised protocol add to their site file. We will update the protocol in the clinical trial registry after the revised protocol is delivered to all the subcenters.

## Discussion

HBV infection remains a major global health concern, as the disease itself and its complications, mainly hepatocellular carcinoma and cirrhosis, caused 887,000 deaths in 2015 alone [26]. Currently, therapeutic drugs for CHB are limited to two types of NAs and interferon. Nucleotide drugs can inhibit virus replication for a long time, but they cannot prevent the synthesis of cccDNA [27], so it is still challenging to achieve clinical cure. Functional cure refers to a continuously negative HBV DNA level and negative conversion or seroconversion of HBsAg, but it can only be realized in a few patients. A systematic evaluation of 34 published studies involving 42,588 patients showed that the annual clearance rate of HBsAg in CHB patients was approximately 1% (including treated and untreated populations) and that in HBeAg-negative patients and HBeAg-positive patients, it was 1.33% (95% CI, 0.76–2.05) and 0.40% (95% CI, 0.25–0.59), respectively [28]. Therefore, lifelong medication is required for most patients. However, long-term treatment with nucleotide drugs has problems that cannot be ignored. Although the rates of resistance to ETV and TDF are low, the likelihood that a patient will develop resistance in the future is unclear. In addition, the high economic burden brought by long-term oral drugs is a vital trade-off factor. The withdrawal of NAs can significantly reduce expenditures for medical care and public health. Additionally, the side effects of NAs cannot be neglected [29], such as the renal toxicity and skeletal toxicity of the first-line therapeutic drug TDF. The new drug TAF bypasses this problem, but its cost is high; thus, the drug has not been widely applied in many countries.

In recent years, some scholars have proposed the concept of immune control, in which the host’s immune system still exerts significant and explicit control over HBV after drug withdrawal; thus, HBV DNA remains at an immeasurable level, and no virology or clinical relapse will occur for a long time after drug withdrawal (beyond 6 months). Therefore, long-term virology inhibition after the withdrawal of nucleotide drugs can be regarded as a possible endpoint of therapy. According to the APASL guidelines, HBeAg-positive patients can discontinue drugs under monitoring by strict follow-up visits after the serological conversion of e-antigen and consolidation therapy for 12 months [22].

Current studies show that the risk of relapse after NA withdrawal is high [30]. A prospective study included 178 CHB patients; 59.5% of the patients suffered virological relapse within 2 years, and 48% suffered a clinical relapse [31]. However, the safety of drug withdrawal for HBeAg-positive patients seems to be higher than that for HBeAg-negative patients. Liu F et al. [32] conducted a long-course observational study on drug withdrawal. A total of 223 patients were included in the study. The results showed that the 10-year relapse rate of HBeAg-positive patients was significantly lower than that of HBeAg-negative patients (30.9% vs. 62.3%). Surprisingly, some studies show that a small number of patients eventually achieve HBsAg clearance after the withdrawal of nucleotide drugs and temporary relapse [33, 34]. A large Taiwan study included a total of 1075 HBeAg-negative patients treated with NAs. Six patients obtained HBsAg clearance during the treatment, and the annual HBsAg clearance rate was approximately 0.15%. A total of 691 patients discontinued NAs according to the APASL guidelines. After a median follow-up of 155 weeks, 42 patients achieved HBsAg clearance, the 6-year cumulative HBsAg clearance rate was 13%, and the annual HBsAg clearance rate was approximately 1.78%. Some patients achieved HBsAg clearance after drug withdrawal and virological relapse [35]. Although there is no unambiguous interpretation for this research result, drug withdrawal does seem possible for some patients. An increasing number of studies have shown that the level of HBsAg during withdrawal is an important factor influencing the relapse rate. A systematic evaluation including 11 studies and 1716 patients showed that regardless of the state of HBeAg during drug withdrawal, the relapse rate in patients with HBsAg level greater than 100 IU/mL was significantly higher than that in patients with HBsAg level under 100 IU/mL. The virological relapse rates of the above two populations were 9.1–19.6% and 31.4–86.8% after drug withdrawal for more than 12 months, and the clinical relapse rates were 15.4–29.4% and 48.1–63.6%, respectively. Moreover, patients with HBsAg level less than 100 IU/ml at drug withdrawal were more likely to achieve spontaneous immune clearance after drug withdrawal [36] than patients with HBsAg levels greater than 100 IU/ml at drug withdrawal. In addition, some scholars have carried out relevant research on other critical markers in the HBV replication cycle. Large and medium surface proteins of HBV (LHBs and MHBs [37], respectively), HBV RNA, and HBcrAg [38, 39] may be serological indicators of cccDNA and have predictive value in the efficacy monitoring of antiviral treatments and the outcome after drug withdrawal. The risk of virological recurrence after drug cessation in patients who have undergone long-term NA therapy will be observed in the current study. It is also our goal to further investigate the criteria for safe drug withdrawal and to explore the predictive factors of relapse. Previous studies have shown that the combination of TCM and NAs can improve HBeAg loss, especially in refractory populations, and that the long-term effect of the combination treatment is more feasible than that of NA monotherapy but does not compromise the safety profile [40]. Interestingly, certain herbal combinations could act as “immune-incubation agents” or “synergists of NAs” in treating HBV infection, particularly in HBeAg-positive CHB patients. This is the first time that a combination of Chinese and Western medicines has been applied to reduce the off-drug recurrence risks in NA-treated populations. The results will provide patients with alternative options, especially in those who have undergone long-term NA treatment and are unwilling to take NAs for life.

## Trial status

The first subject was recruited in March 2019. A total of 490 patients will be subsequently recruited, and the recruitment is currently open. The trial status has been updated in the Chinese clinical trial registry database. Recruitment is expected to end in early 2020. The version number and date of the protocol are v1.0 and December 11, 2018, respectively.

## Data Availability

Data are collected and written on printed forms. And we will share the clinical research data in the system of Chinese Clinical Trials Registry (No. ChiCTR1900021232, 2019/02/02, http://www.chictr.org.cn/showproj.aspx?proj=35297).

## Abbreviations

ALT: Alanine aminotransferase
cccDNA: Closed circular DNA
CHB: Chronic hepatitis B
ECG: Electrocardiogram
ETV: Entecavir
HBeAg: Hepatitis B e antigen
HBsAg: Hepatitis B surface antigen
HBV: Hepatitis B virus
IFN: Interferon
NAs: Nucleos(t)ide
RCT: Randomized controlled trial
RGT: Biejia ruangan
TBIL: Total bilirubin
TCM: Traditional Chinese medicine
WM: Western medicine
TGBXJD: Tiaogan-Buxu-Jiedu
ULN: Upper limit of normal
